# EMR-L has the potential to replace ESD in resecting gastric stromal tumors smaller than 1.0 cm in diameter: A pilot study

**DOI:** 10.1097/MD.0000000000045292

**Published:** 2025-10-24

**Authors:** Mingjie He, Yungang Liu, Lixia Zhang, Yi Cai, Min Xu, Wenwen Xu, Lijuan Xu, Jing Cai, Peipei Luo

**Affiliations:** a Department of Gastroenterology, Wujin People’s Hospital affiliated to JiangSu University, Changzhou, China; b Department of Oncology, Wujin People’s Hospital affiliated to JiangSu University, Changzhou, China; c Department of Internal Medicine, Rulin Town Health Center of Jintan District, Changzhou, China.

**Keywords:** endoscopic ligation assisted mucosalresection, endoscopic mucosal dissection, gastric stromal tumor, hospitalization expenses, length of hospital stay, operation duration

## Abstract

**Background:**

Endoscopic submucosal dissection (ESD), though effective in the treatment of gastric stromal tumor (GST), is limited by difficult procedures, a long learning curve and a high hospital cost. It is of great clinical value to explore new effective, simple, and safe surgical methods.

**Methods:**

The basic data were collected from 25 patients with GSTs < 1.0 cm in diameter who were randomized into endoscopic ligator-assisted mucosal resection (EMR-L) group (n = 12) and ESD group (n = 13). The efficacy in 2 groups was compared in terms of operation duration, cost, complication, length of stay (LOS) and complete resection rate.

**Results:**

The complete resection rate of GST was 100% in both groups. In the EMR-L group, the average operation duration was 16.92 ± 4.76 minutes, the average LOS was 6.12 ± 1.55 days, and the average hospital cost was 17,136.87 ± 2959.80 yuan. In the ESD group, the average operation duration was 46.46 ± 12.27 min, the average LOS was 7.53 ± 2.24 days, and the average hospital cost was 22,760.24 ± 5199.45 yuan.

**Conclusions:**

EMR-L and ESD can achieve the same safety and complete resection rate, but the former has the advantages of simple operation, short operation duration and low hospital cost. EMR-L may serve as a new option for the treatment of GSTs.

## 1. Introduction

Gastrointestinal stromal tumors (GISTs), the most common mesenchymal neoplasm, originate from gastrointestinal interstitial cells of Cajal or their common precursor cells and may emerge in the stomach or the small intestine at any age (median 60–65 years).^[1]^ Patients with a mesenchymal tumors smaller than 2.0 cm in diameter is recommended to be followed up, but repeated endoscopy during a long-term follow-up may exert a heavy financial or psychological stress on the patient. As a result, some specialists in Europe and Japan recommend to remove the histopathologically proven GISTs, even if the tumor is smaller than 2.0 cm.^[2,3]^

Endoscopic submucosal dissection (ESD) is a method proven safe and effective in the treatment of GISTs, with an overall en-bloc resection rate of 92.4% to 100% for submucosal tumor.^[4,5]^ However, its wide use is hindered by difficult procedures and high cost. As suggested, endoscopic ligator-assisted mucosal resection (EMR-L) is feasible for gastric stromal tumor (GST) smaller than 1.0 cm. Its efficacy has been rarely analyzed. In this study including 25 patients, we compared the efficacy of EMR-L and ESD in the treatment of GSTs <1.0 cm.

## 2. Materials and methods

### 2.1. Inclusion and exclusion

Inclusion criteria: age ≥ 18 years; complete clinical data; no coagulation dysfunction; and gastroscopy showing a single GST in the upper part of the body or the fundus of stomach,Patients with GSTs highly suspected by preoperative endoscopic ultrasonography (EUS), all exhibiting lesions with maximum diameters < 1.0 cm on EUS evaluation; (5) patients strongly requesting resection of the lesions by EMR-L or ESD, and providing informed consent; (6) Preoperative histopathological examination confirming the diagnosis of GST. Exclusion criteria: incomplete clinical data; no endoscopic resection; the lesion not completely resected; and allergic to rubber.

### 2.2. Patients

This study was reviewed and approved by the Ethics Committee of Wujin People’s Hospital affiliated to JiangSu University. Of the 123 patients highly suspected of having GSTs based on EUS at Wujin People’s Hospital Affiliated to Jiangsu University from June 2019 to June 2023, a total of 25 patients (14 males [56%] and 11 females [44%], mean 64.08years with GSTs <1.0 cm in diameter were selected and randomly assigned to EMR-L group (n = 12, 48%) and ESD group (n = 13, 52%) (Table 1). The clinical symptoms were upper abdominal fullness, belching, and acid regurgitation, etc. With an average diameter of 7.69 ± 1.28 mm, all the GSTs were located in the gastric fundus or the upper part of the stomach body. Of the 12 patients (48%) receiving EMR-L, one was complicated with postoperative delayed bleeding which was successfully stopped by endoscopy. Of the 13 patients (52%) receiving ESD, one was complicated with intraoperative massive bleeding which could not be controlled by endoscopy, and transferred to surgery for laparoscopic hemostasis. None developed serious abdominal infection. In the EMR-L group were 7 males and 5 females, with an average age of 66.5 (62.75, 72.75) years. In the ESD group were 7 males and 6 females, with an average age of 67 (58, 72) years. There were no significant differences in gender ratio, age and lesion location between the 2 groups (*P* > .05).

**Table 1:** Comparison of general data of 2 groups.

Groups	Gender		Age (yr)	Location		
	Male	Female		Fundus	Body	Antrum
EMR-L	7	5	66.5 (62.75, 72.75)	8	4	0
ESD	7	6	67 (58, 72)	8	5	0
*P* value	.735 (Fisher test)		.764 (Wilcoxon)	.560 (Fisher test)		

EMR = endoscopic ligator-assisted mucosal resection, ESD = endoscopic submucosal dissection.

### 2.3. Outcomes

The outcomes of 2 methods were compared in terms of operation duration, hospital cost, intraoperative perforation, complete resection rate, length of stay (LOS), and serious complications. Complete excision was defined as the horizontal and vertical margins of the lesion pathological tissue being normal gastric tissue. A serious surgical complication was defined as intraoperative and postoperative bleeding, perforation, or infection that was difficult to be controlled by endoscopy or drugs. The general information of the 2 groups of is shown in Table 1.

### 2.4. Materials

The materials used in this study included GIF-Q260J (Olympus Corporation, Japan), EU-ME2(PLUS) Endoscopic ultrasound (Olympus Corporation, Japan), snare (Olympus Corporation, Japan), endoscopic injection needle (Olympus Corporation, Japan), IT knife, dual knife (Olympus Corporation, Japan), hemostatic forceps (Olympus Corporation, Japan), endoscopic titanium clamp (Micro-Tech (Nanjing) Co., Ltd, China) ligation device (MBL-6-F,Wilson-Cook Medical Incorporated, United States), high-frequency electrical cutting device and APC300 argon ion coagulator (ERBE Elektromedizin GmbH, Germany), normal saline, indigocarmine, 1:10 000 adrenalin, and other drugs.

### 2.5. Preoperative preparation

All patients underwent electrocardiogram, macro-biochemistry and coagulation function examinations before surgery. CT and other imaging examinations were completed to rule out the possibility of tumor metastasis. Anticoagulant and antiplatelet drugs such as warfarin, aspirin and clopidogrel were not taken in orally within the week before surgery. After locating the lesions by gastroscopy, ultrasonic gastroscopy was performed. The shape, size, depth, boundary, and internal echo intensity of the lesions were measured to determine their statuses. All patients underwent gastroscopy performed by associate chief physicians, experienced endoscopists with more than 300 cases of ESD surgery experience, or a GIF-260J electronic gastroscope under general anesthesia in the operating room.

### 2.6. Resection of gastric stromal tumor by EMR-L

The ligation device was installed on the gastroscope. The cap of the device was used to target the lesion, and the lesion was fully attracted into the cap of the ligation device and the full visual field was red. The rubber band was released to ligate the lesion, and the mucosa at the bottom of the rubber band was placed with the snare. High-frequency electrical resection was performed until the lesion was completely removed. Hemostatic forceps were used to stop bleeding at the wound, followed by closure with a titanium clamp. The number of titanium clips was determined by the size of the wound.

### 2.7. Resection of gastric stromal tumor by ESD

A mixture of normal saline, Meilan, 1:10,000 epinephrine and sodium hyaluronate was injected in the anterior tumor base and the surrounding mucosa. After the mucosa around the lesion was fully lifted, the mucosa was fully cut off with a dual knife, and the lesion and the submucosal tissue were separated layer by layer until the lesion was completely removed. The dual knife was replaced with an IT knife during the operation if necessary. Hemostatic forceps were used to stop the bleeding at the wound, followed by closure with titanium clips. The number of clips was determined according to the size of the wound.

### 2.8. Statistical analysis

SPSS 20.0 was used for statistical analysis. Measurement data were expressed as mean ± standard deviation (x ± s), count data as component ratio. Between-group comparison was performed by Fisher test or *t* test. *P* < .05 was considered as statistically different.

## 3. Results

### 3.1. Endoscopic characteristics of lesions in 2 groups

**Figure 1.**
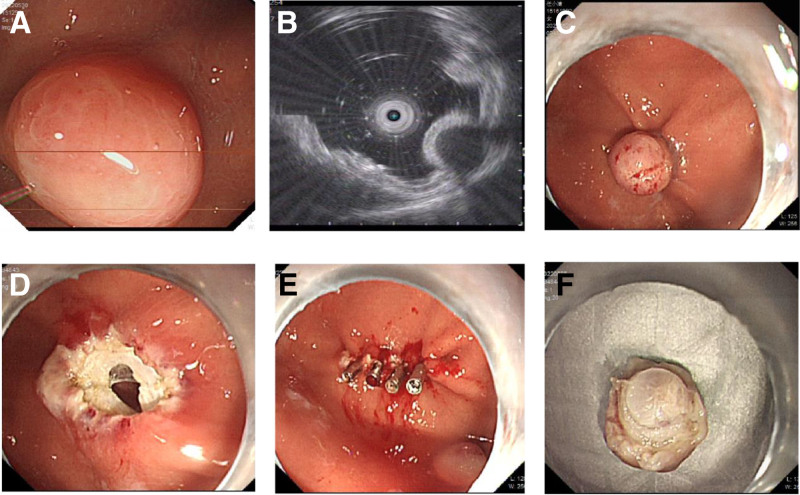
Endoscopic mucosal resection-ligation. (A) White light endoscopic view of gastric stromal tumor. (B) Endoscopic ultrasound view of gastric stromal tumor. (C) Endoscopic view of ligated gastric stromal tumor. (D) Snare resection performed below the ban. (E) Wound closure with titanium clips. (F) A specimen of gastric stromal tumor.

**Figure 2.**
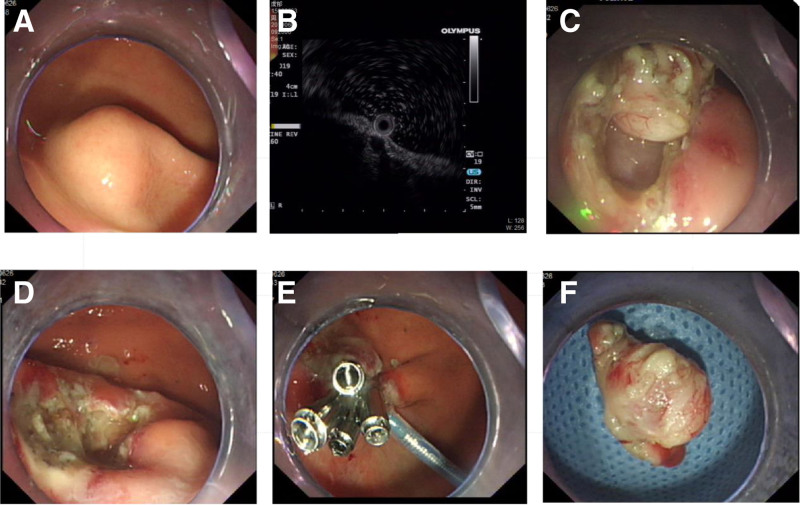
Endoscopic submucosal dissection. (A) White light endoscopic view of gastric stromal tumor. (B) Endoscopic ultrasound view of gastric stromal tumor. (C) Intraoperative wound. (D) Postoperative wound. (E) Wound closure with titanium clips and a nylon loop. (F) A Specimen of gastric stromal tumor.

Gastroscopy showed all the lesions were located in the fundus or the upper part of the stomach body, with diameters <1.0 cm. Most of the lesions were subspherical, hemispherical or hummocic eminences, covered with normal mucosa and containing hard texture. Endoscopic ultrasonography showed uneven echo or hypoecho in the lesions, with a diameter of <1.0 cm. All lesions originated from the musculi propria and grew into the gastric lumen, with clear boundaries free of vascular infiltration. There were no significant differences in tumor location and size between the 2 groups (*P* > .05). The typical endoscopic manifestations of 2 groups are shown in Figure 1 and Figure 2, respectively.

### 3.2. Outcomes

There were no significant differences in intraoperative perforation and serious complication between the 2 groups (*P *> .05). In the EMR-L group, the mean lesion size was 7.13 ± 1.05 mm, all originating from the muscle propria. The tumor was located in the fundus of the stomach in 8 cases, near the fundus of the stomach body in 4 cases. The tumor was completely resected in all the 8 cases, with a complete resection rate of 100%, a mean operation duration of 16.92 ± 4.76 minutes. The average LOS was 6.12 ± 1.55 days, and the average hospital cost was 17,136.87 ± 2959.80 yuan. In the ESD group, the lesions had an average size of 8.04 ± 1.28 mm, all originating from the musculi propria. The tumor was located at the fundus of the stomach in 8 cases, the greater curvature in 3 cases and the lesser curvature in 2 cases. The tumor was complete resected in all the 13 cases, with a complete resection rate of 100%. The mean operation duration was 46.46 ± 12.27 minutes, the mean LOS was 7.53 ± 2.24 days, the average hospital cost was 22,760.24 ± 5199.45 yuan (Table 2).

**Table 2:** Comparison of surgery outcomes in 2 groups.

Groups	Lesion size (mm)	Operation duration (min)	Intraoperative perforation (case)	Serious complication (case)	LOS (d)	Hospital cost (yuan)
EMR-L	7.13 ± 1.05	16.92 ± 4.76	10	0	6.12 ± 1.55	17,136.87 ± 2959.80
ESD	8.04 ± 1.28	46.46 ± 12.27	9	1	7.53 ± 2.24	22,760.24 ± 5199.45
* P* value (*T*-test)	.123	<.001	.465 (Fisher test)	1.000 (Fisher test)	.1467	.003 (Welch *t* test)

EMR = endoscopic ligator-assisted mucosal resection, ESD = endoscopic submucosal dissection.

### 3.3. Pathological results

Postoperative histopathological examination showed that all tumors were mesenchymal (extremely low risk), and presented mitotic images < 5/50HPF, active cell growth, and involvement of muscle lamina proper. Immunohistochemistry showed 20 cases of CD117(+), 20 cases of DOG-1 (+), 17 cases of CD34 (+), S-100 (−), CD57 (−). SMA (−), Desmin (−). All lesions achieved endoscopic *en bloc* resection.

### 3.4. Complications

The main complications were perforation and bleeding in 2 groups. There were 10 (83.33%) patients of intraoperative perforation in the EMR-L group and 9 (69.23%) cases in the ESD group, all successfully clipped with titanium clips (combined with nylon rope if necessary) under the endoscope, followed by fasting, gastrointestinal decompression, inhibition of gastric acid secretion, and prevention of infection after surgery. No clinical manifestations of peritonitis were observed, and the postoperative recovery was ideal. In the EMR-L group, there was no intraoperative and postoperative massive bleeding, with only a small amount of extravasation from some wounds during the operation. In the ESD group, one case exhibited intraoperative massive bleeding, which failed endoscopic hemostasis and necessitated laparoscopic surgery.

### 3.5. Follow-up

Postoperative gastroscopy was regularly performed to follow-up all the patients. Up to now, no recurrence has been observed, with a longest follow-up of 4 years.

## 4. Discussion

GISTs, first proposed by Professor Mazur in 1983, are a group of tumors originating from the mesenchymal tissue of the gastrointestinal tract, with an annual global prevalence of 13 per 100,000 people.^[6]^ GISTs can occur in men and women at any age, especially those aged over 60 years.^[7]^ Consistently in the present study, the incidence of GSTs was similar between men and women. About 60% of GSTs develop in the stomach (more common in the fundus than in the body and antrum) and 30% in the small intestine^[8,9]^, this result is consistent with our statistics. It has been reported that the median tumor diameter at diagnosis is 6 cm.^[10]^ Gastroscopy in wide use has detected more GSTs in recent years.

As suggested by Joensuu et al^[11]^, malignancy is suspected if a GIST has a diameter larger than 3 cm, an irregular border, ulcers on the surface, and a non-oval shape. According to the National Comprehensive Cancer Network guidelines, all GISTs have malignant potential, and GISTs with a diameter larger than 2 cm should be resected. For patients with GISTs <2 cm in diameter, endoscopic or endoscopic follow-up has previously been recommended. However, long-term follow-up and repeated endoscopic examinations will bring serious negative influences, such as mental distress.^[12]^ Therefore, resection is recommended even if the tumor diameter is smaller than 2.0 cm.^[2,3,13]^ Endoscopic surgery now serves as a safe method to resect submucosal tumors of the digestive tract, including ESD, submucosaltunnel endoscopic resection (STER), endoscopic submucosal excavation, and endoscopic full-thickness resection.

A retrospective cohort study reported that a 6.0 cm GST should also be completely removed under endoscopy.^[14]^ Liu et al^[14]^ showed that in resecting GSTs smaller than 5.0 cm in diameter, endoscopy achieved the same safety and effectiveness as laparoscopic or open surgery. Ye et al^[4]^ applied STER to remove GST, and no residue or recurrence during the follow-up suggested that this technique is safe and feasible for the treatment of GST smaller than 3 cm, therefore, ESD is very safe for the treatment of GSTs smaller than 1.0 cm. Endoscopic surgery, such as ESD and STER, requires deftness of operators and a long learning curve, which restricts its wide application. Besides, it needs a large wound surface, a high hospital cost and a long LOS. The present study provides evidence that EMR-L may replace ESD in the treatment of GSTs.

Meanwhile, Sun et al^[15]^ first reported that endoscopic band ligation was safe and effective in the treatment of GSTs smaller than 1.2 cm. Meng et al^[16]^ showed similar outcomes of endoscopic band ligation in removing GSTs smaller than 1.5 cm, but with higher recurrence rate than ESD. Oono et al^[17]^ reported the effectiveness of EMR-L in treating neuroendocrine tumors smaller than 1.0 cm, suggesting that this method is as simple as ESD and achieve a high complete resection rate as EMR does. The neuroendocrine tumor is often located in the gastrointestinal wall at a level similar to that of GST, so we speculate that this approach is feasible for the treatment of GSTs with a diameter smaller than 1.0 cm.

Our results showed no statistical difference between the EMR-L group and the ESD group in age of onset, location of the lesion in the gastric cavity, gender ratio and lesion diameter. In addition, a 100% complete resection was achieved in both groups, and the operation duration in the EMR-L group was significantly shorter than that in the ESD group. This is mainly because the gastroscopy in the EMR-L group was relatively simpler. Although 83.33% of gastroscopy perforations occurred and the perforation rate was significantly higher in the EMR-L group, the wound surface was smaller and more regular. Therefore, it is relatively simpler to clamp the wound with titanium clips. Compared with ESD group, EMR-L group had a shorter LOS and a lower hospital cost.

With new endoscopic suture techniques and materials, perforation and bleeding during and after endoscopic surgery can be handled well under endoscopy. In terms of complications, no delayed perforation or serious infection occurred in the 2 groups. In this series, intraoperative perforation occurred in 19 of 25 patients (76%). Upon perforation detection, we reduced gastric insufflation to prevent significant pneumoperitoneum and subsequent intra-abdominal hypertension. However, this reduction in insufflation compromised endoscopic visualization of the defect margins, thereby increasing the technical difficulty of closure.Existing evidence indicates that post-ESD gastric defects smaller than 2 cm can be reliably closed using standard titanium clips, while through-the-scope clip closure becomes technically challenging for defects exceeding 2 cm. To address this clinical challenge, researchers have developed various advanced closure techniques including endoscopic hand-suturing,^[18]^ combination of endoloop and through-the-scope clips,O-ring ligation closure,^[19]^ the “accordion fold” method^[20]^ and knot-assisted loop suturing methods^[21]^ for large post-ESD defects.In our study cohort, given the relatively small lesion sizes and correspondingly limited perforation dimensions, all cases achieved successful primary closure.

One patient in the ESD group had gastrointestinal bleeding that was difficult to be controlled by endoscope during the operation and was successfully stopped by laparoscopic surgery. We still hold that EMR-L has a higher safety than ESD. In performing EMR-L, the blood vessels, especially those in the gastric serous layer, cannot be observed and pretreated in advance. Therefore, the patient may face a risk of intraoperative and postoperative bleeding, so we performed hot biopsy forceps to stop bleeding on the wound after surgery.

The present study has some limitations. First, a larger sample size should be based on to evaluate the surgical safety in our future study. In addition, due to the small size of the ligation device cap, the lesion would better be operated within a range of 1.3 cm, Despite studies of Sun,^[15]^ Meng,^[16]^ and Meng^[22]^ showed that EMR-L could encase 1.3 cm or even 2.0 cm GSTs, our preliminary experiment also found that some GSTs larger than 1.0 cm could not be completely covered in rubber bands. Sometimes, due to the thick gastric mucosa and submucosa or edema of the gastric wall, it is difficult to inhale the ligation device even if the tumor is small, which greatly limits the application of this surgery.

## 5. Conclusions

In conclusion, compared with ESD, EMR-L achieves a higher safety and a complete resection rate for GST smaller than 1.0 cm in diameter, with simpler procedures, shorter operation duration and lower hospital cost. This technique is expected to be a new method for the treatment of GSTs. However, due to the small sample size and short follow-up time in this study, future studies are recommended to verify its safety and efficacy.

## Author contributions

**Conceptualization:** Peipei Luo.

**Data curation:** Mingjie He, Peipei Luo.

**Formal analysis:** Mingjie He, Wenwen Xu.

**Investigation:** Yungang Liu, Lixia Zhang, Peipei Luo.

**Methodology:** Lixia Zhang, Peipei Luo.

**Writing – original draft:** Yungang Liu, Yi Cai, Jing Cai.

**Writing – review & editing:** Min Xu, Lijuan Xu.

## References

[1] Joensuu H Hohenberger P Corless CL. Gastrointestinal stromal tumour. Lancet. 2013;382:973–83. 23623056 10.1016/S0140-6736(13)60106-3

[2] Demetri GD, von Mehren M Antonescu CR. NCCN Task Force report: update on the management of patients with gastrointestinal stromal tumors. J Natl Compr Canc Netw. 2010;8 Suppl 2:S1–41; quiz S42–44. 10.6004/jnccn.2010.0116 PMC4103754 20457867

[3] Nishida T Hirota S Yanagisawa A. Clinical practice guidelines for gastrointestinal stromal tumor (GIST) in Japan: English version. Int J Clin Oncol. 2008;13:416–30. 18946752 10.1007/s10147-008-0798-7

[4] Ye LP Zhang Y Mao XL Zhu L-H Zhou X Chen J-Y. Submucosal tunneling endoscopic resection for small upper gastrointestinal subepithelial tumors originating from the muscularis propria layer. Surg Endosc. 2014;28:524–30. 24013472 10.1007/s00464-013-3197-8

[5] He G Wang J Chen B. Feasibility of endoscopic submucosal dissection for upper gastrointestinal submucosal tumors treatment and value of endoscopic ultrasonography in pre-operation assess and post-operation follow-up: a prospective study of 224 cases in a single medical center. Surg Endosc. 2016;30:4206–13. 26823060 10.1007/s00464-015-4729-1

[6] Nishida T Blay JY Hirota S Kitagawa Y Kang YK. The standard diagnosis, treatment, and follow-up of gastrointestinal stromal tumors based on guidelines. Gastric Cancer. 2016;19:3–14. 26276366 10.1007/s10120-015-0526-8 PMC4688306

[7] Quiroz HJ Willobee BA Sussman MS. Pediatric gastrointestinal stromal tumors-a review of diagnostic modalities. Transl Gastroenterol Hepatol. 2018;3:54. 30225388 10.21037/tgh.2018.07.08 PMC6131159

[8] Søreide K Sandvik OM Søreide JA Giljaca V Jureckova A Bulusu VR. Global epidemiology of gastrointestinal stromal tumours (GIST): A systematic review of population-based cohort studies. Cancer Epidemiol. 2016;40:39–46. 26618334 10.1016/j.canep.2015.10.031

[9] Miettinen M Lasota J. Gastrointestinal stromal tumors: pathology and prognosis at different sites. Semin Diagn Pathol. 2006;23:70–83. 17193820 10.1053/j.semdp.2006.09.001

[10] Mantese G. Gastrointestinal stromal tumor: epidemiology, diagnosis, and treatment. Curr Opin Gastroenterol. 2019;35:555–9. 31577561 10.1097/MOG.0000000000000584

[11] Joensuu H. Risk stratification of patients diagnosed with gastrointestinal stromal tumor. Hum Pathol. 2008;39:1411–9. 18774375 10.1016/j.humpath.2008.06.025

[12] Mekky MA Yamao K Sawaki A. Diagnostic utility of EUS-guided FNA in patients with gastric submucosal tumors. Gastrointest Endosc. 2010;71:913–9. 20226456 10.1016/j.gie.2009.11.044

[13] Puchalski CM. Spirituality in the cancer trajectory. Ann Oncol. 2012;23:49–55. 22628416 10.1093/annonc/mds088

[14] Liu Z Zeng Z Ouyang S. Comparison among endoscopic, laparoscopic, and open resection for relatively small gastric gastrointestinal stromal tumors (<5 cm): a Bayesian network meta-analysis. Front Oncol. 2021;11:672364. 34912700 10.3389/fonc.2021.672364 PMC8667731

[15] Sun S Ge N Wang C. Endoscopic band ligation of small gastric stromal tumors and follow-up by endoscopic ultrasonography. Surg Endosc. 2007;21:574–8. 17103278 10.1007/s00464-006-9028-4

[16] Meng Y Cao C Song S Li Y Liu S. Endoscopic band ligation versus endoscopic submucosal dissection and laparoscopic resection for small gastric stromal tumors. Surg Endosc. 2016;30:2873–8. 26490768 10.1007/s00464-015-4571-5

[17] Oono Y Shinmura K Hori K. Endoscopic submucosal resection using a ligation device without injection for duodenal neuroendocrine tumors. Surg Endosc. 2019;33:2008–14. 30604268 10.1007/s00464-018-06642-5

[18] Goto O Sasaki M Akimoto T. Endoscopic hand-suturing for defect closure after gastric endoscopic submucosal dissection: a pilot study in animals and in humans. Endoscopy. 2017;49:792–7. 28561197 10.1055/s-0043-110668

[19] Nishiyama N Kobara H Kobayashi N. Efficacy of endoscopic ligation with O-ring closure for prevention of bleeding after gastric endoscopic submucosal dissection under antithrombotic therapy: a prospective observational study. Endoscopy. 2022;54:1078–84. 35213923 10.1055/a-1782-3448 PMC9613440

[20] Ikenoyama Y Katsurahara M Tanaka K. Complete closure of a large mucosal defect (100 mm) after gastric endoscopic submucosal dissection, using the “accordion fold” method. Endoscopy. 2022;54:E892–3. 35750076 10.1055/a-1860-1528 PMC9735413

[21] Liu WH Li ZS Wang D. Endoscopic closure of a large gastric mucosal defect after endoscopic submucosal dissection using a novel endoscopic string-with-knotter suture device. Endoscopy. 2022;54:E19–21. 33598898 10.1055/a-1348-0645

[22] Meng R Ni M Ren W. Comparison of modified cap-assisted endoscopic mucosal resection and endoscopic submucosal dissection in treating intraluminal gastric gastrointestinal stromal tumor (≤20 mm). Clin Transl Gastroenterol. 2023;14:e00589. 37019655 10.14309/ctg.0000000000000589 PMC10299766

